# Invasive pulmonary aspergillosis among ICU patients with COVID-19, influenza, and preexisting host factors: a nationwide cohort study

**DOI:** 10.1186/s12879-026-13250-5

**Published:** 2026-04-06

**Authors:** Nakyung Jeon, Mihyun Park, Moon Seong Baek, Tae Wan Kim, Won-Young Kim

**Affiliations:** 1 College of Pharmacy and Research Institute for Drug Development, Pusan National University, Busan, Republic of Korea; 2 Division of Pulmonary and Critical Care Medicine, Department of Internal Medicine, Chung-Ang University Hospital, Chung-Ang University College of Medicine, Seoul, Republic of Korea

**Keywords:** COVID-19, Human influenza, Intensive care units, Invasive pulmonary aspergillosis, Mechanical ventilation, Risk factors

## Abstract

**Background:**

Previous studies on the association between coronavirus disease 2019 (COVID-19) and invasive pulmonary aspergillosis (IPA) were limited by their single-center nature and small patient population. Whether aspergillosis risk is related to COVID-19 or other factors remains unclear. This study aimed to determine whether COVID-19 among intensive care unit (ICU) patients is associated with an increased incidence of IPA compared with that among individuals with influenza or preexisting host factors (control).

**Methods:**

This was a nationwide study performed using the Korean National Health Insurance Service database. A cohort of 28,089 patients with COVID-19 (2020–2022), 9993 individuals with influenza (2015–2019), and 11,807 control patients (2015–2019) were analyzed. Odds ratios (ORs) and 95% confidence intervals (CIs) of outcomes were calculated between the propensity score-matched groups.

**Results:**

After matching, patients with COVID-19 were paired with 9649 patients with influenza (mean [SD] age, 72.7 [15.1] years vs. 72.7 [14.5] years; men, 53.3% vs. 52.2%) and 5709 control patients (mean [SD] age, 71.8 [12.0] years vs. 72.2 [11.1] years; men, 70.0% vs. 72.2%). The COVID-19 group was not associated with an increased risk of IPA compared to the observations in the influenza (OR, 1.20; 95% CI 0.97–1.48) or the control (OR, 1.06; 95% CI 0.89–1.26) group. Consistent findings were observed for patients receiving high-flow nasal cannula or mechanical ventilation. The risk factors for IPA included male sex, chronic pulmonary disease, chronic liver disease, admission to higher-volume hospitals, renal replacement therapy, and length of hospital stay; COVID-19 was not implicated (COVID-19 vs. influenza; adjusted OR, 1.22; 95% CI 0.97–1.54 and COVID-19 vs. control; adjusted OR, 1.17; 95% CI 0.97–1.42). In the COVID-19 group, corticosteroid use for ≥ 7 days was associated with an increased risk of IPA, regardless of the dosage.

**Conclusions:**

ICU patients with COVID-19 were not at an increased risk of IPA compared to those with influenza or preexisting host factors. COVID-19 may not predispose to IPA.

**Supplementary Information:**

The online version contains supplementary material available at 10.1186/s12879-026-13250-5.

## Background

The influenza virus causes direct injury to the airway epithelium and ciliary dysfunction, leading to immune dysregulation and tissue invasion by *Aspergillus* [1, 2]. Coronavirus disease 2019 (COVID-19) may also lower mucociliary clearance and increase aspergillosis risk [3]. Additionally, the lymphocyte count is decreased among patients with COVID-19 [4], which is a known risk factor for invasive fungal disease in patients with hematological malignancies [5].

The clinical manifestations of COVID-19 are comparable to those of severe influenza [6]. As influenza-associated pulmonary aspergillosis (IAPA) is a frequent complication among patients with influenza admitted to the intensive care unit (ICU) [6], patients with severe COVID-19 may also be susceptible to invasive pulmonary aspergillosis (IPA). Conversely, COVID-19 and influenza differ based on the epithelial damage degree and the involved cytokines [7]. These may result in profound heterogenous manifestations of aspergillosis among patients with COVID-19. The association between COVID-19 and aspergillosis has been explored [8]; however, most reports of COVID-19-associated pulmonary aspergillosis (CAPA) were from single-center studies with a small patient population. Moreover, whether the incidence of CAPA is owing to COVID-19 or other factors remains unclear [9]. Dexamethasone has become the standard treatment for severe COVID-19 [10]; however, the data explaining whether a higher-dose and/or longer corticosteroid regimen or other immunomodulatory drugs increase the risk of CAPA are lacking.

To address the current knowledge gaps, the primary aim of this study was to analyze the risk of IPA among ICU patients with COVID-19, influenza, and preexisting host factors (control) using the Korean National Health Insurance Service (NHIS) database. Patients with preexisting host factors were determined according to the consensus definitions by the European Organization for Research and Treatment of Cancer and the Mycoses Study Group (EORTC-MSG) [9]. Subgroup analyses were conducted in patients receiving high-flow nasal cannula or mechanical ventilation, given that ventilation increases bronchial inflammation and reduces mucociliary clearance, which may exacerbate the risk of aspergillosis [3]. Similarly, we aimed to determine whether host factors, disease severity, or steroids (their dosage, duration of treatment, and type) were associated with the risk of IPA among these patients.

## Methods

### Study design and data source

This nationwide population-based study retrieved data from the NHIS database to construct COVID-19, influenza, and control cohorts. The study protocol for the analysis of the de-identified patient data was exempted from review by the Institutional Review Board of Chung-Ang University (1041078-20230621-HR-168). This study complied with the Strengthening the Reporting of Observational Studies in Epidemiology guidelines [11].

The NHIS database comprises reimbursement claims from all citizens in Korea except for healthcare beneficiaries for veterans [12]. The data provide detailed inpatient and outpatient information, including demographics, insurance premiums, employment status, disabilities, primary and secondary diagnoses, prescriptions, procedures, discharge outcome, and date of death. The database is also linked to the Korean nationwide COVID-19 registry designed by the Korea Disease Control and Prevention Agency to confirm COVID-19 diagnoses and vaccination cases. The diagnostic codes were based on the International Classification of Diseases, 10th Revision (ICD-10). All prescribed drugs were identified using Anatomical Therapeutic Chemical codes and the Korean Health Insurance Review and Assessment Service charge codes. Data were extracted by an independent technician at the NHIS center.

### Study population

For COVID-19 cases, adult (age ≥ 18 years) patients with confirmed COVID-19 who were admitted to the ICU between October 2020 and October 2022 were included. The predominant SARS-CoV-2 variants circulating in Korea were wild type and Alpha from October 2020 to June 2021, Delta from July 2021 to December 2021, and Omicron from January 2022 to October 2022.

Adult (age ≥ 18 years) patients with influenza or preexisting host factors who were admitted to the ICU were constructed as the historical control groups to (1) compare the risk of IPA with those of COVID-19 patients and (2) assess the sequelae specific to COVID-19. Influenza cases were identified using the ICD-10 codes for influenza (J09, J10, or J11) between January 2015 and December 2019. Patients with preexisting host factors were determined between January 2015 and December 2019. Host factors included hematological malignancy, allogenic stem-cell transplant, solid organ transplant, the use of corticosteroids at ≥ 20 mg/day (prednisolone-equivalent) for ≥ 3 weeks in the past 60 days, treatment with T-cell immunosuppressants (such as calcineurin inhibitors, tumor necrosis factor blockers, lymphocyte-specific monoclonal antibodies, and immunosuppressive nucleoside analogs) during the past 90 days, treatment with inhibitors of B-cell receptor pathway, inherited severe immunodeficiency, or acute graft-versus-host disease (Additional file 1: Table S1) [9]. These host factors were converted into a single binary variable when defining the study group.

For patients with multiple ICU admissions, only the first admission was included. The exclusion criteria were age < 18 years, hospitalization before or after the inclusion period, death or discharge within the first 2 days of hospitalization, pregnancy or related conditions, palliative care, or cardiac arrest. All patients were followed until death or 180 days after the hospitalization day.

### Invasive pulmonary aspergillosis

IPA was defined according to the ICD-10 code (B44.0) or the use of at least one dose of antifungal agents (Additional file 1: Table S2) during or within 1–30 days after the index hospitalization. Isavuconazole was excluded because the drug is not currently reimbursed by the NHIS. Similarly, echinocandins were excluded, as they are used as first-line regimens for invasive candidiasis. Patients who had been prescribed antifungal agents for over 2 weeks within 6 months before the hospitalization and those diagnosed with mucormycosis (B46) during admission were excluded.

### Data collection, definitions, and outcomes

The baseline patient characteristics included age, sex, Charlson Comorbidity Index [13] defined on the basis of claim codes within 1 year before admission (Additional file 1: Table S3), immunosuppression, income level derived from data on insurance premiums [14], and hospital size. The type of organ dysfunction was identified using ICD-10 codes (Additional file 1: Table S4). Corticosteroid use was defined as at least one dose of intravenous dexamethasone, hydrocortisone, or methylprednisolone during hospitalization. All doses were converted to methylprednisolone equivalents [15], and the daily dose and total duration of use were calculated. Vasopressor use was defined as the administration of norepinephrine, epinephrine, vasopressin, dopamine, or dobutamine during the hospitalization. Procedure codes were used to retrieve cases that involved supplemental oxygen, high-flow nasal cannula, mechanical ventilation, renal replacement therapy, and extracorporeal membrane oxygenation.

The primary outcome was IPA. The secondary outcomes included 30- and 180-day mortality, ventilator days, tracheostomy, and ICU and hospital lengths of stay.

### Statistical analysis

Data were presented as the mean (standard deviation, SD) or median (interquartile range, IQR) for continuous variables and as numbers (percentages) for categorical variables. Outcome data were presented as the median of all paired differences with 95% confidence intervals (CIs).

Propensity score (PS) matching using logistic regression was conducted to adjust for differences in the baseline variables when comparing the COVID-19 and influenza groups, as well as the COVID-19 and control groups [16]. For the logistic regression PS model, COVID-19 was used as the dependent variable, and the independent variables were the baseline covariates listed in Table 1. The PS for COVID-19 diagnosis was estimated for each patient. Subsequently, matching was performed in a 1:1 ratio without the replacement of patients in the COVID-19 group vs. the influenza group, as well as the COVID-19 group vs. the control group, using nearest-neighbor matching based on an 8:1-digit greedy matching algorithm [17]. Covariate balance before and after matching was evaluated using standardized mean differences (SMDs). To account for the paired nature of the PS-matched data, multivariable conditional logistic regression models were constructed to compute the adjusted odds ratios (ORs) and 95% CIs of the outcomes associated with COVID-19. Separate logistic regression models were constructed to identify the risk factors for IPA. Independent variables were the baseline covariates listed in Table 1 and study groups.

**Table 1:** Baseline characteristics of intensive care unit patients in the matched cohort

Characteristics	COVID-19 (*n* = 9649)	Influenza (*n* = 9649)	SMD	COVID-19 (*n* = 5709)	Control^a^ (*n* = 5709)	SMD
Age, mean (SD), y	72.7 (15.1)	72.7 (14.5)	< 0.001	71.8 (12.0)	72.2 (11.1)	0.03
Sex, No. (%)			0.02			0.05
Male	5147 (53.3)	5036 (52.2)		3995 (70.0)	4123 (72.2)	
Female	4502 (46.7)	4613 (47.8)		1714 (30.0)	1586 (27.8)	
Comorbidities, No. (%)						
Diabetes	4909 (50.9)	4839 (50.2)	0.02	3738 (65.5)	3704 (64.9)	0.01
Hypertension	6528 (67.7)	6570 (68.1)	0.01	4214 (73.8)	4210 (73.7)	< 0.001
Myocardial infarction	671 (7.0)	653 (6.8)	0.01	527 (9.2)	540 (9.5)	0.01
Congestive heart failure	2679 (27.8)	2631 (27.3)	0.01	2309 (40.4)	2280 (39.9)	0.01
Cerebrovascular disease	2998 (31.1)	2932 (30.4)	0.02	1840 (32.2)	1826 (32.0)	0.01
Chronic pulmonary disease	5070 (52.5)	5264 (54.6)	0.04	3335 (58.4)	3404 (59.6)	0.02
Chronic liver disease	3661 (37.9)	3641 (37.7)	< 0.001	3201 (56.1)	3212 (56.3)	< 0.001
Chronic kidney disease	1241 (12.9)	1219 (12.6)	0.01	1333 (23.3)	1295 (22.7)	0.02
Malignancy	1728 (17.9)	1641 (17.0)	0.02	4248 (74.4)	4178 (73.2)	0.03
Charlson Comorbidity Index, mean (SD)	3.6 (3.0)	3.5 (2.8)	0.03	6.4 (3.5)	6.7 (3.6)	0.08
Immunosuppression, No. (%)^b^	2303 (23.9)	2231 (23.1)	0.02	5698 (99.8)	5700 (99.8)	< 0.001
Income level, No. (%)						
Q1 (lowest)	2609 (27.0)	2627 (27.2)	< 0.001	1404 (24.6)	1424 (24.9)	0.01
Q2	2356 (24.4)	2234 (23.2)	0.03	1446 (25.3)	1433 (25.1)	0.01
Q3	2461 (25.5)	2529 (26.2)	0.02	1494 (26.2)	1464 (25.6)	0.01
Q4 (highest)	2223 (23.0)	2259 (23.4)	0.01	1365 (23.9)	1388 (24.3)	0.01
Hospital size, No. (%)						
< 500 beds	4590 (47.6)	4730 (49.0)	0.03	1954 (34.2)	1881 (32.9)	0.03
500–1000 beds	4329 (44.9)	4247 (44.0)	0.02	2807 (49.2)	2833 (49.6)	0.01
≥ 1000 beds	730 (7.6)	672 (7.0)	0.02	948 (16.6)	995 (17.4)	0.02
Organ dysfunction, No. (%)						
Cardiovascular	4692 (48.6)	4570 (47.4)	0.03	3631 (63.6)	3568 (62.5)	0.02
Respiratory	8445 (87.5)	8531 (88.4)	0.03	5222 (91.5)	5183 (90.8)	0.02
Neurologic	1003 (10.4)	922 (9.6)	0.03	578 (10.1)	544 (9.5)	0.02
Hematologic	1071 (11.1)	878 (9.1)	0.06	1151 (20.2)	1045 (18.3)	0.05
Hepatic	176 (1.8)	151 (1.6)	0.02	199 (3.5)	185 (3.2)	0.02
Renal	1972 (20.4)	1882 (19.5)	0.02	1548 (27.1)	1537 (26.9)	< 0.001
Metabolic	261 (2.7)	269 (2.8)	0.01	190 (3.3)	187 (3.3)	< 0.001
Corticosteroids, No. (%)	4475 (46.4)	4132 (42.8)	0.07	3668 (64.2)	3484 (61.0)	0.07
Daily dose, median (IQR), mg^c^	53 (45–82)	65 (33–125)	0.02	53 (48–93)	63 (35–125)	< 0.001
Total days of use, median (IQR)	6 (3–10)	4 (1–9)	0.07	8 (4–14)	4 (1–12)	0.098
Vasopressor use, No. (%)	4528 (46.9)	4411 (45.7)	0.02	3536 (61.9)	3481 (61.0)	0.02
Oxygen therapy						
No oxygen	1221 (12.7)	1136 (11.8)	0.03	493 (8.6)	532 (9.3)	0.02
Supplemental oxygen	4380 (45.4)	4738 (49.1)	0.08	1831 (32.1)	1949 (34.1)	0.04
High-flow nasal cannula	750 (7.8)	679 (7.0)	0.02	762 (13.3)	729 (12.8)	0.02
Mechanical ventilation	3298 (34.2)	3096 (32.1)	0.03	2623 (45.9)	2499 (43.8)	0.05
Neuromuscular blocking agents, No. (%)	2256 (23.4)	2000 (20.7)	0.06	2425 (42.5)	2381 (41.7)	0.02
Renal replacement therapy, No. (%)	748 (7.8)	651 (6.7)	0.04	813 (14.2)	800 (14.0)	0.01
ECMO, No. (%)	32 (0.3)	36 (0.4)	0.01	25 (0.4)	14 (0.2)	0.03

*COVID-19* coronavirus disease 2019, *ECMO* extracorporeal membrane oxygenation, *IQR* interquartile range, *SD* standard deviation, *SMD* standardized mean difference

^a^ Patient with one of the following: hematological malignancy; allogenic stem-cell transplant; solid organ transplant; use of corticosteroids at ≥ 20 mg/day (prednisolone-equivalent) for ≥ 3 weeks in the past 60 days; treatment with T-cell immunosuppressants, such as calcineurin inhibitors, tumor necrosis factor blockers, lymphocyte-specific monoclonal antibodies, and immunosuppressive nucleoside analogs, during the past 90 days; treatment with inhibitors of B-cell receptor pathway; inherited severe immunodeficiency; or acute graft-versus-host disease

^b^ Immunosuppression included malignancy, human immunodeficiency virus infection, organ transplant, or immunosuppressive therapy

^c^ Methylprednisolone doses and converted doses of dexamethasone and hydrocortisone

Baseline, outcome, and risk factor analyses were performed among high-risk subgroups. The high-risk subgroup was defined as that comprising patients requiring high-flow nasal cannula or mechanical ventilation. High-flow nasal cannula and mechanical ventilation were selected given that ventilation may increase bronchial inflammation and decrease mucociliary clearance, which may worsen the risk of aspergillosis [3]. Three sensitivity analyses were performed to assess the robustness of the results. First, analyses were performed to ascertain the associations between the dosage of, duration of treatment with, and type of steroids and IPA. The cut-offs for the daily steroid dose and the total days of steroid use were determined based on the median values of the study patients. Second, the associations between IPA and interleukin (IL)-6 receptor antagonist (tocilizumab) and Janus kinase inhibitor (baricitinib) were evaluated to assess whether immunomodulatory drugs affect the development of CAPA. Third, to assess the effects of the vaccine and Omicron on CAPA incidence, the outcomes were assessed through stratification according to the vaccination status before admission and the study period (non-Omicron vs. Omicron).

The dataset had no missing values. The results of the secondary analyses should be considered hypothesis-generating owing to the potential for type I error caused by multiple comparisons. All statistical analyses were performed using the SAS software (version 9.4; SAS Institute, Cary, NC, USA). All tests were two-tailed, and differences were considered statistically significant at *P* < 0.05.

## Results

### Patient characteristics

**Fig. 1:**
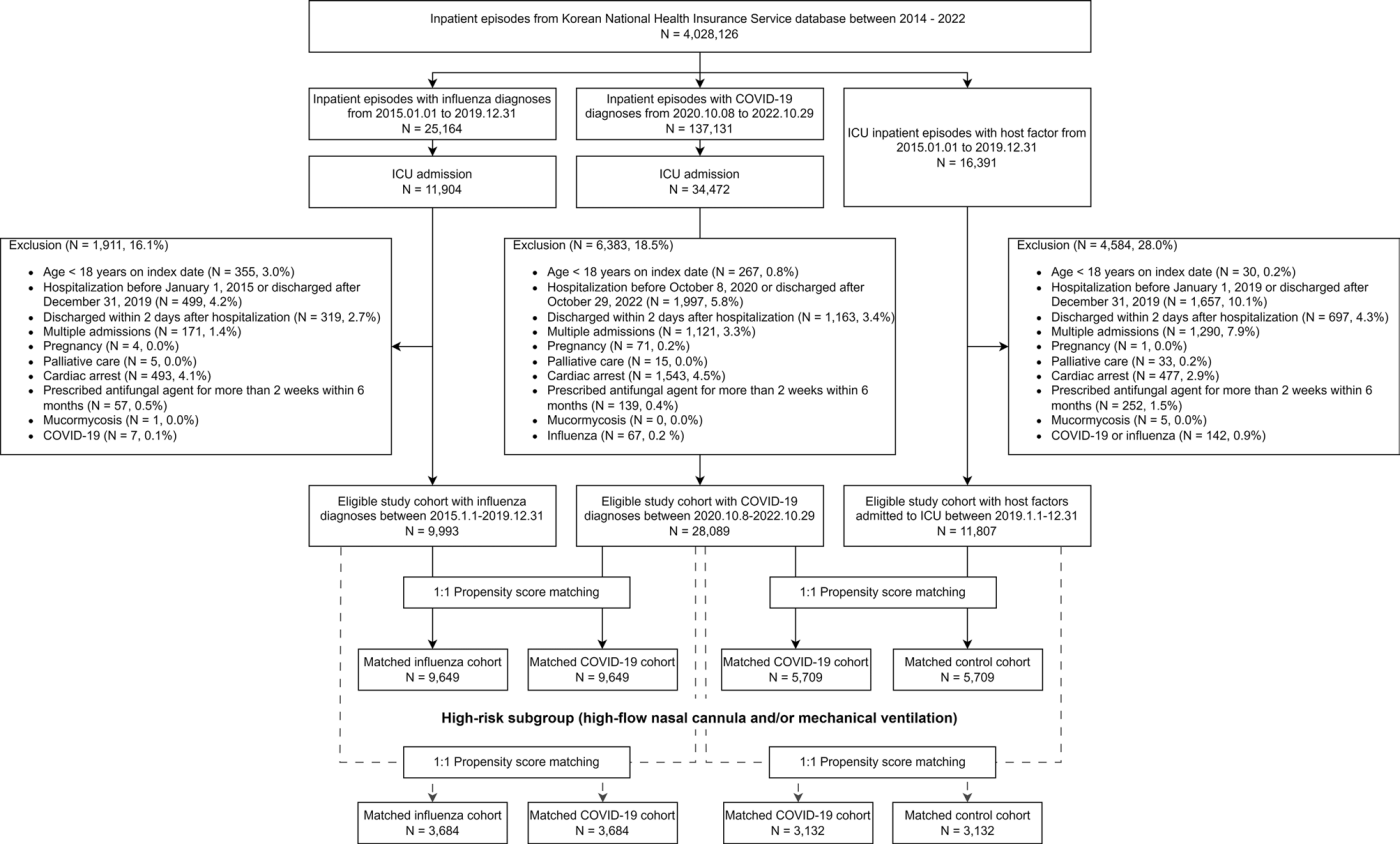
Flowchart describing the selection process of patients for the COVID-19, influenza, and control cohorts. *COVID-19* coronavirus disease 2019, *ICU* intensive care unit

After removing some participants based on the exclusion criteria, 28,089 out of 34,472 (81.5%) ICU-admitted patients with COVID-19, 9993 out of 11,904 (83.9%) with influenza, and 11,807 out of 16,391 (72.0%) controls were included in the primary analysis (Fig. 1). The baseline characteristics of the three cohorts are presented in Additional file 1: Table S5. Because control patients were selected according to the EORTC-MSG criteria, the group was more likely to comprise males and exhibit a high Charlson Comorbidity Index (mean, 5.8), immunosuppression, and admission to a higher-volume hospital. The patients in the COVID-19 group had high cardiovascular, hematologic, hepatic, and renal dysfunctions, along with high frequencies of vasopressor, high-flow nasal cannula, mechanical ventilation, neuromuscular blocking agent, and renal replacement therapy uses. Moreover, patients in the COVID-19 group were more likely to receive corticosteroids with longer duration of use; however, the daily dose was not significantly different compared with those in the influenza and control groups.

### Primary and secondary outcomes in the COVID-19 vs. influenza and COVID-19 vs. control comparisons

After PS matching, 9649 patients with COVID-19 (mean [SD] age, 72.7 [15.1] years; men, 53.3%) were successfully paired with patients with influenza (mean [SD] age, 72.7 [14.5] years; men, 52.2%; Fig. 1; Table 1). The PS-matched COVID-19 (mean [SD] age, 71.8 [12.0] years; men, 70.0%) and control (mean [SD] age, 72.2 [11.1] years; men, 72.2%) groups of 5709 patients each were also acquired. The SMDs between the groups were < 0.10, indicating well-balanced baseline characteristics (Table 1). The COVID-19 group was more likely to receive corticosteroids for longer durations, although the differences between the groups were not statistically significant.

In the primary outcome, patients with COVID-19 exhibited similar incidence of IPA (2.0% vs. 1.6%; difference, 0.3%; 95% CI − 0.1% to 0.7%; *P* = 0.09), with an OR of 1.20 (95% CI 0.97–1.48) to that of individuals with influenza (Table 2). Among secondary outcome variables, the COVID-19 group demonstrated significantly higher 30- and 180-day mortality rates and lengths of ICU and hospital stay than did the influenza group; however, ventilator days and tracheostomy rates were significantly higher in the influenza group. The incidences of IPA were 4.8% and 4.6% (difference, 0.2%; 95% CI − 0.5% to 1.0%; *P* = 0.54) among patients in the COVID-19 and control groups, respectively (Table 2), and the risk was also not significantly higher in the COVID-19 group (OR, 1.06; 95% CI 0.89–1.26). The 30-day mortality, ventilator days, tracheostomy rates, and lengths of ICU and hospital stay were significantly higher among patients with COVID-19 than in the control patients.

**Table 2:** Primary and secondary outcomes

Outcomes	COVID-19 (*n* = 9649)	Influenza (*n* = 9649)	Difference (95% CI)^a^	*P* value^b^	Odds ratio (95% CI)
**Primary outcome**					
Invasive pulmonary aspergillosis, No. (%)	190 (2.0)	159 (1.6)	0.3 (–0.1 to 0.7)	0.09	1.20 (0.97–1.48)
**Secondary outcomes**					
30-day mortality, No. (%)	2071 (21.5)	1557 (16.1)	5.3 (4.2 to 6.4)	< 0.001	1.42 (1.32–1.53)
180-day mortality, No. (%)	3548 (36.8)	2951 (30.6)	6.2 (4.9 to 7.5)	< 0.001	1.32 (1.24–1.40)
Ventilator days, median (IQR)	6 (2–12) [*n* = 3298]	6 (3–13) [*n* = 3096]	0 (–1 to 0)	0.01	
Tracheostomy, No. (%)	397 (4.1)	474 (4.9)	–0.8 (–1.4 to − 0.2)	0.01	0.83 (0.73–0.95)
Length of stay, median (IQR), days					
ICU	5 (2–10)	4 (2–9)	0 (0 to 0)	< 0.001	
Hospital	17 (9–32)	16 (9–28)	1 (1 to 1)	< 0.001	

Outcomes	COVID-19 (*n* = 5709)	Control^c^ (*n* = 5709)	Difference (95% CI)^a^	*P* value^b^	Odds ratio (95% CI)
**Primary outcome**					
Invasive pulmonary aspergillosis, No. (%)	275 (4.8)	261 (4.6)	0.2 (–0.5 to 1.0)	0.54	1.06 (0.89–1.26)
**Secondary outcomes**					
30-day mortality, No. (%)	1672 (29.3)	1575 (27.6)	1.7 (0 to 3.4)	0.04	1.09 (1.00–1.18)
180-day mortality, No. (%)	2948 (51.6)	2983 (52.3)	–0.6 (–2.4 to 1.2)	0.51	0.98 (0.91–1.05)
Ventilator days, median (IQR)	7 (3–14) [*n* = 2623]	4 (2–11) [*n* = 2499]	1 (1 to 2)	< 0.001	
Tracheostomy, No. (%)	414 (7.3)	309 (5.4)	1.8 (0.9 to 2.7)	< 0.001	1.37 (1.17–1.59)
Length of stay, median (IQR), days					
ICU	5 (2–10)	3 (1–9)	1 (0 to 1)	< 0.001	
Hospital	20 (11–36)	19 (11–34)	1 (0 to 1)	< 0.001	

*CI* confidence interval, *COVID-19* coronavirus disease 2019, *ICU* intensive care unit, *IQR* interquartile range

^a^ Median of all paired differences between the study groups

^b^ *P* values were calculated using the Wilcoxon rank sum test for continuous variables and the chi-square test for categorical variables

^c^ Patient with one of the following: hematological malignancy; allogenic stem-cell transplant; solid organ transplant; use of corticosteroids at ≥ 20 mg/day (prednisolone-equivalent) for ≥ 3 weeks in the past 60 days; treatment with T-cell immunosuppressants, such as calcineurin inhibitors, tumor necrosis factor blockers, lymphocyte-specific monoclonal antibodies, and immunosuppressive nucleoside analogs, during the past 90 days; treatment with inhibitors of B-cell receptor pathway; inherited severe immunodeficiency; or acute graft-versus-host disease

### Risk factors for IPA

Among ICU patients with COVID-19 and those with influenza, the independent predictors of IPA were male sex, chronic pulmonary disease, chronic liver disease, immunosuppression, admission to higher-volume hospitals, corticosteroid use, neuromuscular blocking agent use, renal replacement therapy, and length of hospital stay (Table 3). In the COVID-19 vs. control comparison, the independent predictors of IPA were male sex, chronic pulmonary disease, admission to higher-volume hospitals, corticosteroid use, renal replacement therapy, and length of hospital stay (Table 3). The total duration of steroid use was an independent predictor of IPA, while the cumulative steroid dose was not. In either case, COVID-19 was not significantly associated with an increased risk of IPA (COVID-19 vs. influenza; adjusted OR, 1.22; 95% CI 0.97–1.54 and COVID-19 vs. control; adjusted OR, 1.17; 95% CI 0.97–1.42).

**Table 3:** Risk factors for invasive pulmonary aspergillosis

Variable	COVID-19 vs. Influenza (*n* = 9649 vs. 9649)		COVID-19 vs. Control^a^ (*n* = 5709 vs. 5709)	
	Adjusted odds ratio (95% CI)^b^	*P* value	Adjusted odds ratio (95% CI)^b^	*P* value
Age, y				
18–39	1 (reference)		1 (reference)	
40–64	0.88 (0.51–1.53)	0.65	0.98 (0.37–2.67)	0.98
≥ 65	0.77 (0.44–1.33)	0.34	0.73 (0.27–1.96)	0.52
Female sex	0.74 (0.58–0.94)	0.01	0.50 (0.37–0.66)	< 0.001
Comorbidities				
Diabetes	1.13 (0.88–1.46)	0.33	1.07 (0.87–1.33)	0.51
Hypertension	0.79 (0.60–1.02)	0.07	0.86 (0.69–1.07)	0.17
Myocardial infarction	0.79 (0.47–1.32)	0.37	0.68 (0.45–1.04)	0.07
Congestive heart failure	0.77 (0.57–1.05)	0.10	0.77 (0.61–0.97)	0.02
Cerebrovascular disease	0.77 (0.58–1.02)	0.07	0.77 (0.61–0.98)	0.03
Chronic pulmonary disease	1.47 (1.14–1.89)	< 0.001	1.37 (1.11–1.68)	< 0.001
Chronic liver disease	1.42 (1.06–1.91)	0.02	1.04 (0.83–1.31)	0.72
Chronic kidney disease	1.09 (0.77–1.54)	0.61	0.99 (0.77–1.27)	0.93
Malignancy	1.01 (0.67–1.53)	0.95	0.97 (0.74–1.28)	0.85
Immunosuppression^c^	1.71 (1.19–2.45)	< 0.001	N/A	
Hospital size				
< 500 beds	1 (reference)		1 (reference)	
500–1000 beds	1.80 (1.30–2.47)	< 0.001	2.40 (1.68–3.43)	< 0.001
≥ 1000 beds	4.55 (3.13–6.60)	< 0.001	4.91 (3.36–7.18)	< 0.001
No. of organ dysfunctions				
1	1 (reference)		1 (reference)	
2	0.91 (0.41–2.03)	0.81	0.77 (0.38–1.58)	0.48
3	1.40 (0.38–5.15)	0.61	0.78 (0.25–2.41)	0.66
≥ 4	1.54 (0.23–10.55)	0.66	0.45 (0.09–2.33)	0.34
Corticosteroids	2.62 (1.85–3.72)	< 0.001	4.34 (2.86–6.59)	< 0.001
Cumulative dose^d^	1.00 (1.00–1.00)	0.07	1.00 (1.00–1.00)	0.56
Total days of use	1.03 (1.02–1.04)	< 0.001	1.03 (1.02–1.03)	< 0.001
Vasopressor use	1.01 (0.36–2.84)	0.98	2.38 (0.72–7.90)	0.16
Neuromuscular blocking agents	1.34 (1.02–1.75)	0.03	0.99 (0.79–1.24)	0.92
Renal replacement therapy	1.80 (1.20–2.71)	0.01	1.64 (1.18–2.27)	< 0.001
ECMO	1.59 (0.73–3.47)	0.25	0.74 (0.28–1.95)	0.55
Length of hospital stay	1.00 (1.00–1.01)	< 0.001	1.00 (1.00–1.01)	< 0.001
COVID-19	1.22 (0.97–1.54)	0.09	1.17 (0.97–1.42)	0.10

*CI* confidence interval, *COVID-19* coronavirus disease 2019, *ECMO* extracorporeal membrane oxygenation, *N/A* not applicable

^a^ Patient with one of the following: hematological malignancy; allogenic stem-cell transplant; solid organ transplant; use of corticosteroids at ≥ 20 mg/day (prednisolone-equivalent) for ≥ 3 weeks in the past 60 days; treatment with T-cell immunosuppressants, such as calcineurin inhibitors, tumor necrosis factor blockers, lymphocyte-specific monoclonal antibodies, and immunosuppressive nucleoside analogs, during the past 90 days; treatment with inhibitors of B-cell receptor pathway; inherited severe immunodeficiency; or acute graft-versus-host disease

^b^ Adjusted for the baseline characteristics listed in Table 1

^c^ Immunosuppression included malignancy, human immunodeficiency virus infection, organ transplant, or immunosuppressive therapy

^d^ Methylprednisolone doses and converted doses of dexamethasone and hydrocortisone

### High-risk subgroup analyses

After a 1:1 covariate matching, 3684 patients with COVID-19 receiving high-flow nasal cannula or mechanical ventilation were successfully paired with patients with influenza (Fig. 1). The PS-matched high-risk COVID-19 and control groups of 3132 patients each were also obtained. Admission to a higher-volume hospital, organ dysfunction, corticosteroids, neuromuscular blocking agents, and organ support were more prevalent in these groups than in the primary cohort (Additional file 1: Table S6). Patients with COVID-19 exhibited similar incidence of IPA to those in the influenza (3.7% vs. 3.3%; difference, 0.4%; 95% CI − 0.5% to 1.2%; *P* = 0.41) and control (7.9% vs. 7.0%; difference, 0.9%; 95% CI − 0.4% to 2.2%; *P* = 0.19) groups; however, the incidences were higher than that in the primary cohort (Additional file 1: Table S7). The results of secondary outcomes were consistent with those in the primary analysis. Among high-risk patients with COVID-19 vs. those with influenza, the independent predictors of IPA were admission to higher-volume hospitals, corticosteroid use, and total duration of steroid use (Additional file 1: Table S8). In the high-risk COVID-19 vs. control comparison, male sex, chronic pulmonary disease, admission to higher-volume hospitals, corticosteroid use, total duration of steroid use, renal replacement therapy, and length of hospital stay were the independent predictors of IPA. Notably, COVID-19 was significantly associated with an increased risk of IPA (adjusted OR, 1.28; 95% CI 1.04–1.57).

### Associations of the dosage of, duration of treatment with, and type of steroids with IPA

**Fig. 2:**
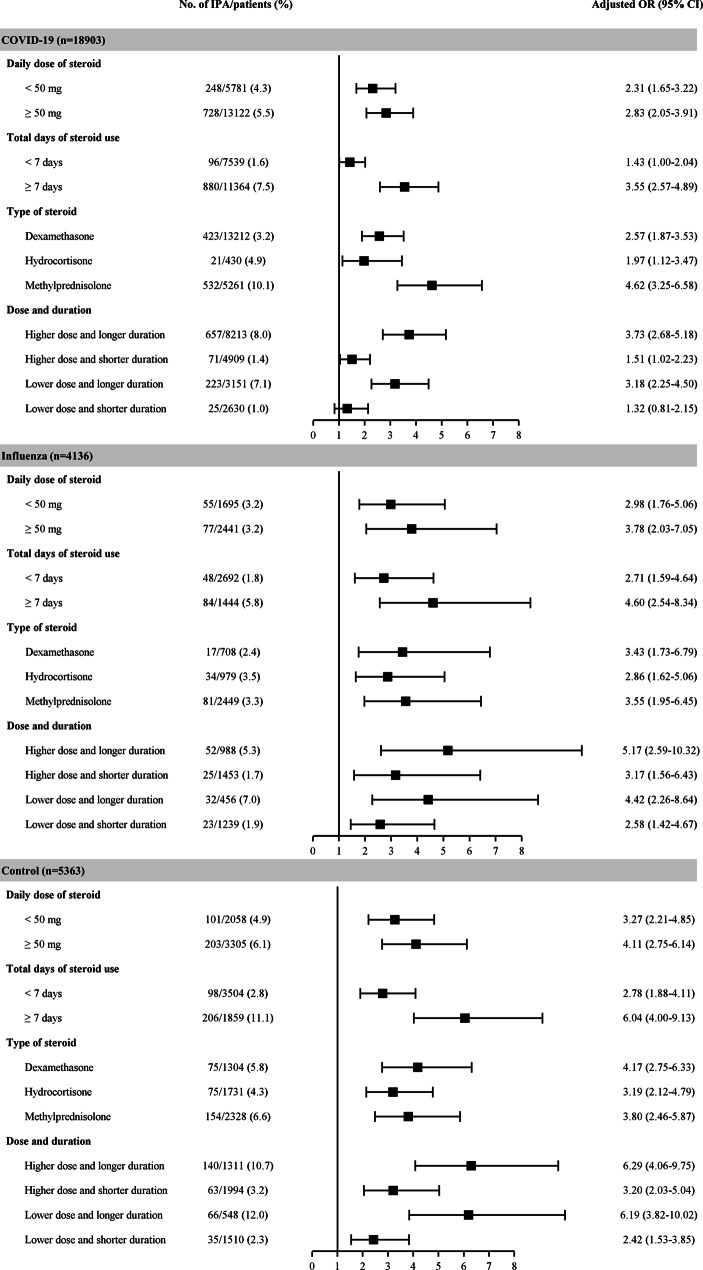
Associations between the dosage of, duration of treatment with, and type of steroids and IPA in the COVID-19, influenza, or control group. The numbers and percentages of patients who acquired infection according to each risk factor and the resulting odds ratios. The odds ratios were adjusted for the baseline covariates listed in Table 1. *CI* confidence interval, *COVID-19* coronavirus disease 2019, *IPA* invasive pulmonary aspergillosis, *OR* odds ratio

In ICU patients with COVID-19, corticosteroid use (regardless of the dosage) for ≥ 7 days was significantly associated with an increased risk of IPA (adjusted OR, 3.55; 95% CI 2.57–4.89; Fig. 2). No significant interaction was observed with the steroid type; however, patients treated with methylprednisolone showed a higher OR (adjusted OR, 4.62; 95% CI 3.25–6.58). Similarly, no significant interaction was observed with any variable among patients with influenza. Similar to that in the COVID-19 group patients, the risk of IPA was significantly high in the control patients who received corticosteroids for ≥ 7 days, regardless of the dosage (adjusted OR, 6.04; 95% CI 4.00–9.13). The primary and secondary outcomes stratified according to the daily dose (≥ 50 vs. < 50 mg) and duration (≥ 7 vs. < 7 days) of steroids are presented in Additional file 1: Table S9. For the three cohorts, the incidence of IPA, mortality rates, ventilator days, tracheostomy rates, and lengths of stay were significantly higher among patients treated with higher/lower doses and longer duration of steroids.

### Risk factors for CAPA

After adjusting for confounding factors, tocilizumab use (adjusted OR, 1.48; 95% CI 1.20–1.83) and baricitinib use (adjusted OR, 2.47; 95% CI 1.93–3.15) were the independent predictors of CAPA among ICU patients with COVID-19 (Additional file 1: Table S10). Immunosupression, admission to higher-volume hospitals, total duration of steroid use, vasopressor use, and renal replacement therapy were also associated with a higher risk of CAPA.

### Effects of the vaccine and Omicron on CAPA incidence

Among the unmatched, influenza-matched, and control-matched COVID-19 cohorts, the incidence of CAPA was lower in the group of vaccinated patients than in the unvaccinated group (Additional file 1: Table S11). CAPA incidence was lower in the Omicron-dominant era than in the non-Omicron period among the three cohorts.

## Discussion

Our PS analysis of ICU-admitted patients with COVID-19 revealed a similar incidence of IPA to that in those with influenza or preexisting host factors. The findings were consistent for patients receiving high-flow nasal cannula or mechanical ventilation. Various demographic and clinical factors were the independent predictors of IPA. However, COVID-19 was not an independent predictor of IPA. The total duration of steroid use increased the risk of IPA, while the cumulative steroid dose did not. Finally, the incidence of CAPA was low in vaccinated patients and during Omicron dominance.

Retrospective case series and prospective studies have reported high incidence rates (20–30%) of IPA among patients with COVID-19 receiving mechanical ventilation [18–20]. Conversely, some studies reported lower incidence rates (3–5%) in similar populations [21, 22]. These findings corroborate the 3–7% incidence rates of high-risk patients with COVID-19 in the current study. Several reasons may explain the large variations in CAPA incidences among critically ill patients. First, overestimating the incidence of CAPA is possible owing to challenges in distinguishing colonization or contamination from infection. Moreover, CAPA incidence decreased from 10% to 4% when the cases were re-examined by an independent committee [23]. Second, previous studies involved various criteria for CAPA [24, 25], complicating outcome comparison. Third, there might be a “center effect” in which CAPA incidence rates vary depending on the level of hospitals participating in the study. Among ICU patients with COVID-19, influenza, and preexisting host factors, admission to a higher-volume hospital was associated with an increased risk of IPA, including those receiving high-flow nasal cannula or mechanical ventilation. Fourth, there are geographical and temporal differences in COVID-19 epidemiology. For example, the 7.9% IPA incidence in the high-risk COVID-19 group (preexisting host factors: 7.0%) corroborates the 9.1% incidence reported in a nationwide multicenter cohort study in Korea [26]. The incidence of CAPA in this study was subsequently decreased during the pandemic when vaccinated patients and the Omicron variant were predominant. The 30- and 180-day mortality rates of patients with CAPA were 31.8% and 69.1%, respectively (data not shown). These corroborate the 40–50% mortality rates of CAPA in previous cohort studies [27, 28].

Early studies demonstrated that only a few patients with CAPA had traditional EORTC-MSG host factors [29]. In severe cases, there are clear similarities between IAPA and CAPA, including proinflammatory response, acute respiratory distress syndrome, and sepsis [6]. Thus, it was reasonable to suggest that, as with IAPA, SARS-CoV-2 infection itself is a major risk factor for CAPA. Indeed, COVID-19 was independently associated with an increased risk of IPA in high-risk patients. However, a study in the vaccination era demonstrated a significant association between CAPA and EORTC-MSG host factors [30]. Furthermore, a recent meta-analysis revealed other host and treatment factors, such as diabetes, cerebrovascular disease, chronic obstructive pulmonary disease (COPD), chronic liver disease, and renal replacement therapy, to be associated with CAPA incidence [8]. The findings are consistent with those of the current study. Structural lung damage and impaired ciliary function in patients with COPD may increase the risk of aspergillosis. These effects would be amplified among patients requiring respiratory support; however, high-flow nasal cannula or mechanical ventilation was not a risk factor for IPA. Data about the impact of liver disease on the development of CAPA are lacking; however, patients with cirrhosis have an increased likelihood of acquiring IAPA owing to immune dysfunction [31]. Renal replacement therapy is also a relevant risk factor for IPA in critically ill patients with influenza [32]. Finally, male sex was identified as a risk factor for IPA, as females induce stronger cellular and humoral immune reactions [33].

Corticosteroid use is a known immunologic risk factor for IAPA [9]. Systemic corticosteroids impair the antifungal host response by inhibiting a specific form of phagocytosis called LC3-associated phagocytosis [34]. After the positive results of the RECOVERY trial [10], corticosteroids are universally used as standard care for severe COVID-19; however, their risk of increasing CAPA incidence remains partially assessed. Several meta-analyses revealed an association between high-dose corticosteroids and CAPA incidence [8, 35]. However, the question remains whether a certain duration or type of steroid administration increases CAPA risk, as detailed descriptions of these factors were missing in most studies. The incidence of IPA was high among patients treated with corticosteroids for ≥ 7 days, even with low daily dose. Conversely, patients who received corticosteroids for < 7 days, albeit with a high daily dose, demonstrated a lower incidence of IPA. These corroborate the finding of a cohort study that steroid administration (at any dose) for > 3 weeks was a risk factor for CAPA [22]. Another study showed a higher proportion of hydrocortisone administration in patients with CAPA than in those without CAPA [36]. In this study, a high risk of IPA was observed among patients receiving methylprednisolone in the COVID-19 group. The risk for CAPA could be increased by anti-IL-6 receptor therapy [37]. Tocilizumab or baricitinib use was associated with a high risk of IPA in patients with COVID-19, consistent with the findings of previous studies [8, 38].

The primary strengths of the current study include its large cohort of the entire Korean population, which enabled various PS and secondary analyses. Previous studies could not fully adjust the confounding factors and were limited by discrepancies regarding the associations between CAPA and host and treatment factors owing to the small patient population [8]. In contrast to previous studies, this study observed the occurrence of IPA for 30 days after hospitalization to include late-onset fungal infection. Furthermore, extending the study period to later in the pandemic enabled the evaluation of the impact of immunomodulatory drugs, such as tocilizumab and baricitinib, on CAPA development and the assessment of CAPA incidence in the vaccination and Omicron periods.

However, this study has some limitations. First, its retrospective design precludes making causal inferences regarding the associations between COVID-19, influenza, preexisting host factors, and outcomes. Although the patients were matched for potential confounding factors in the PS dataset, other variables may not have been included. The inverse probability of treatment weighting would have allowed more patients to be retained in the analysis [39]. However, the method was not used because extreme weights increase variance and reduce balance between covariates. Second, the diagnoses of IPA were based on the ICD-10 or the initiation of antifungal therapy. ICD-10 codes can lack specificity, and it is unclear whether a recorded case represents true IPA or simple colonization. The criteria for suspecting IPA, the investigation process for suspected cases, and mycological methods could have differed between the centers. Additionally, antifungal agents may have been used for prophylactic or empirical purposes. Patients diagnosed with IPA but did not receive antifungal agents might not have been included. Thus, there is a risk of both overdiagnosis and underdiagnosis. Nevertheless, a recent multicenter study demonstrated a considerable degree of agreement between CAPA by consensus criteria and operation definition [40]. Third, the elevated risk of CAPA may have been underestimated by comparing with control patients who were likely to have a higher baseline risk of IPA. However, the immunosuppression covariate used for PS matching closely reflects the EORTC-MSG host factors, which were meticulously balanced between the COVID-19 and control groups. Fourth, the reason for ICU admission in COVID-19, influenza, or control patients may not have been respiratory failure. This limitation can be alleviated by subgroup analyses of patients receiving high-flow nasal cannula or mechanical ventilation. Fifth, there is a possibility of misclassification bias because the analysis did not involve isavuconazole. However, this bias is likely to be minimal, as alternative first-line antifungals (e.g., voriconazole) are more commonly used under the NHIS. Sixth, vital signs and laboratory values were not included in the database; however, diagnoses, prescriptions, and procedures were used as surrogates for disease severity. In this study, neutropenia could not be defined as one of the host factors. Seventh, the data on the timing of ICU admission and IPA were unavailable because of the lack of timestamps. Thus, whether the period between COVID-19 or influenza diagnosis and CAPA or IAPA incidence is associated with outcomes could not be assessed. Lastly, all ICUs included were in Korea, preventing the generalizability of the present findings.

## Conclusions

Patients with COVID-19 were not more vulnerable to IPA than those diagnosed with influenza or with preexisting host factors. The findings were consistent for severe conditions where patients require high-flow nasal cannula or mechanical ventilation. COVID-19 may not be a risk factor for IPA; however, the risk is associated with underlying host factors or therapies. Current data could aid in the risk stratification of CAPA, particularly in the absence of typical risk factors. In critically ill patients with COVID-19, corticosteroids could be used for the recommended duration. However, discontinuing treatment should be considered, regardless of dosage, when there is no evidence of hyperinflammation or suspicion of secondary infections.

## Supplementary Information

Below is the link to the electronic supplementary material.

Supplementary Material 1: Additional file 1: Table S1 Coding for the case definition of preexisting host factors. Table S2 Types and codes of antifungal agents. Table S3 Comorbidities based on the Charlson Comorbidity Index. Table S4 ICD-10-based classification of organ dysfunction. Table S5 Baseline characteristics of intensive care unit patients in the unmatched cohort. Table S6 Baseline characteristics of high-risk subgroup in the matched cohort. Table S7 Primary and secondary outcomes of high-risk subgroup. Table S8 Risk factors for invasive pulmonary aspergillosis in the high-risk subgroup. Table S9 Primary and secondary outcomes according to steroid dose and duration. Table S10 Risk factors for COVID-19-associated pulmonary aspergillosis. Table S11 Incidence of CAPA according to the vaccination status and study period

## Data Availability

All data generated or analyzed during this study are included in this published article and its supplementary information files.

## Abbreviations

CAPA: COVID-19-associated pulmonary aspergillosis
CI: Confidence interval
COPD: Chronic obstructive pulmonary disease
COVID-19: Coronavirus disease 2019
EORTC-MSG: The European Organization for Research and Treatment of Cancer and the Mycoses Study Group
IAPA: Influenza-associated pulmonary aspergillosis
ICD: Internation Classification of Diseases
ICU: Intensive care unit
IPA: Invasive pulmonary aspergillosis
IQR: Interquartile range
NHIS: National Health Insurance Service
OR: Odds ratio
PS: Propensity score
SD: Standard deviation
SMD: Standardized mean difference
