# Supplementary material for: Invasive pulmonary aspergillosis among ICU patients with COVID-19, influenza, and preexisting host factors: a nationwide cohort study
Source: BMC Infect Dis. 2026 Apr 6;26:960. doi: 10.1186/s12879-026-13250-5 (PMC13182017; doi:10.1186/s12879-026-13250-5)
Supplement: Supplementary file 1 — Supplementary Material 1: Additional file 1: Table S1 Coding for the case definition of preexisting host factors. Table S2 Types and codes of antifungal agents. Table S3 Comorbidities based on the Charlson Comorbidity Index. Table S4 ICD-10-based classification of organ dysfunction. Table S5 Baseline characteristics of intensive care unit patients in the unmatched cohort. Table S6 Baseline characteristics of high-risk subgroup in the matched cohort. Table S7 Primary and secondary outcomes of high-risk subgroup. Table S8 Risk factors for invasive pulmonary aspergillosis in the high-risk subgroup. Table S9 Primary and secondary outcomes according to steroid dose and duration. Table S10 Risk factors for COVID-19-associated pulmonary aspergillosis. Table S11 Incidence of CAPA according to the vaccination status and study period [file 12879_2026_13250_MOESM1_ESM.docx]

**Table S1** Coding for the case definition of preexisting host factors

| Variable | ICD-10, EDI, or HIRA charge codes |
| --- | --- |
| Hematological malignancy | C81, C82, C83, C84, C85, C86, C88, C90, C91, C92, C93, C94, C95, C96 |
| Allogenic stem-cell transplant | X5020, X5051, X5061–X5064, X5111–X5115, X5120, X5131–X5137, X6001–X6008 |
| Solid organ transplant | R3275, R3280, Q8040–Q8050, Q8061, Q8062, Q8080, Q8101–Q8103, Q8121–Q8123, Q8131, Q8140–Q8150 |
| Corticosteroids (oral) |  |
| Deflazacort | 140801ATB |
| Dexamethasone | 141901ATB, 141903ATB, 141904ATB |
| Betamethasone | 116401ATB, 296900ATB |
| Hydrocortisone | 170901ATB, 170906ATB |
| Methylprednisolone | 193302ATB, 193305ATB |
| Prednisolone | 217001ATB, 217003ASY, 217004ASY, 217034ASY, 217030ASY, 217035ASY |
| Triamcinolone | 243201ATB, 243202ATB, 243203ATB |
| Fludrocortisone | 160201ATB |
| Corticosteroids (intravenous) |  |
| Dexamethasone | 142001BIJ, 142201BIJ, 142202BIJ, 142030BIJ, 142230BIJ, 142232BIJ, 142233BIJ |
| Betamethasone | 116502BIJ, 116530BIJ |
| Hydrocortisone | 171201BIJ, 171202BIJ |
| Methylprednisolone | 193501BIJ, 193502BIJ, 193601BIJ, 193602BIJ, 193603BIJ, 193604BIJ, 193530BIJ, 193531BIJ |
| Prednisolone | 217302BIJ |
| Triamcinolone | 243301BIJ, 243303BIJ, 243305BIJ, 243336BIJ, 243335BIJ, 243337BIJ |
| Calcineurin inhibitors |  |
| Cyclosporine | 139201ACS, 139204ACS, 194701ACS, 194701ALQ, 194702ACS, 194730ALQ, 194731ALQ, 194703ACS, 139202BIJ, 139230BIJ |
| Tacrolimus | 234201ACH, 234203ACH, 234204ACH, 234205ACR, 234206ACR, 234207ACR, 234201ATB, 234204ATB, 234203ATB, 234208ATB, 234202BIJ, 234230BIJ |
| Tumor necrosis factor blockers |  |
| Etanercept | 455801BIJ, 455802BIJ, 455830BIJ, 455831BIJ, 455803BIJ |
| Infliximab | 383501BIJ, 383502BIJ |
| Adalimumab | 488401BIJ, 488430BIJ, 488431BIJ, 488433BIJ |
| Golimumab | 621201BIJ, 621202BIJ, 621230BIJ, 621231BIJ, 621232BIJ |
| Lymphocyte-specific monoclonal antibodies |  |
| Rituximab | 422601BIJ, 422602BIJ, 422630BIJ, 422631BIJ, 422603BIJ, 422632BIJ |
| Obinutuzumab | 628901BIJ |
| Inotuzumab ozogamicin | 676501BIJ |
| Daratumumab | 667101BIJ, 667102BIJ |
| Nivolumab | 638401BIJ, 638402BIJ, 638403BIJ |
| Pembrolizumab | 639001BIJ |
| Durvalumab | 676901BIJ, 676902BIJ |
| Avelumab | 678801BIJ |
| Atezolizumab | 657701BIJ |
| Brentuximab | 624501BIJ |
| Immunosuppressive nucleoside analogues |  |
| Mercaptopurine | 190601ATB |
| Cladribine | 134801BIJ, 134830BIJ, 134801ATB |
| Fludarabine | 160102ATB, 160101BIJ |
| Clofarabine | 614301BIJ, 614330BIJ |
| Cytarabine | 139601BIJ, 139602BIJ, 139603BIJ, 139604BIJ, 139605BIJ, 139609BIJ, 139633BIJ, 139637BIJ, 139630BIJ, 139631BIJ, 139632BIJ, 139634BIJ, 139635BIJ, 139636BIJ, 139638BIJ |
| 5-fluorouracil | 161401BIJ, 161402BIJ, 161404BIJ, 161430BIJ, 161431BIJ, 161432BIJ |
| Tegafur | 309600ACE, 309600AGN, 309700ACH, 364100AGN |
| Gemcitabine hydrochloride | 452400ACH, 452500ACH, 164901BIJ, 164902BIJ, 164903BIJ, 164930BIJ, 164931BIJ, 164932BIJ |
| Capecitabine | 122701ATB, 122702ATB |
| Azacitidine | 484301BIJ, 484302BIJ |
| Decitabine | 495602BIJ |
| Azathioprine | 112401ATB, 112402ATB |
| Doxifluridine | 149301ACH, 149302ACH |
| Enocitabine | 151901BIJ |
| Inhibitors of B-cell receptor pathway |  |
| Ibrutinib | 628101ACH |
| Venetoclax | 680601ATB, 680602ATB, 680603ATB |
| Belimumab | 642701BIJ, 642702BIJ |
| Inherited severe immunodeficiency | D71, D824, D810, D811, D812, D819 |
| Acute graft-versus-host disease | T860 |

*EDI* Electronic Data Interchange, *HIRA* Health Insurance Review and Assessment Service, *ICD* International Classification of Diseases

**Table S2** Types and codes of antifungal agents

| Variable | ATC codes | HIRA charge codes |
| --- | --- | --- |
| **Voriconazole (oral)** |  |  |
| Voriconazole 200 mg | J02AC03 | 456501ATB |
| **Voriconazole (intravenous)** |  |  |
| Voriconazole 200 mg | J02AC03 | 456501BIJ |
| **Posaconazole** |  |  |
| Posaconazole 40 mg | J02AC04 | 566201ASS |
| Posaconazole 4.2 g | J02AC04 | 566230ASS |
| Posaconazole 0.1 g | J02AC04 | 566202ATE |
| **Itraconazole (oral)** |  |  |
| Itraconazole 100 mg | J02AC02 | 179101ACH |
| Itraconazole 100 mg | J02AC02 | 179101ATB |
| Itraconazole 10 mg | J02AC02 | 179102ALQ |
| Itraconazole 200 mg | J02AC02 | 179104ATB |
| Itraconazole 1.5 g | J02AC02 | 179131ALQ |
| **Itraconazole (intravenous)** |  |  |
| Itraconazole 250 mg | J02AC02 | 179103BIJ |
| Itraconazole 0.25 g | J02AC02 | 179130BIJ |
| **Amphotericin B** |  |  |
| Amphotericin B 50 mg | A01AB04 | 108302BIJ |
| Amphotericin B (colloidal) 100 mg | A01AB04 | 108401BIJ |
| Amphotericin B (colloidal) 50 mg | A01AB04 | 108402BIJ |
| Amphotericin B (liposomal) 50 mg | A01AB04 | 108501BIJ |

*ATC* Anatomical Therapeutic Chemical, *HIRA* Health Insurance Review and Assessment Service

**Table S3** Comorbidities based on the Charlson Comorbidity Index^a^

| Variable | ICD-10 codes |
| --- | --- |
| **Charlson Comorbidity Index** |  |
| Myocardial infarction | I21, I22, I252 |
| Congestive heart failure | I099, I110, I130, I132, I255, I420, I425, I426, I427, I428, I429, I43, I50 |
| Peripheral vascular disease | I70, I71, I731, I738, I739, I771, I790, I792, K551, K558, K559, Z958, Z959 |
| Cerebrovascular disease | G45, G46, I60, I61, I62, I63, I64, I65, I66, I67, I68, I69, H340 |
| Dementia | F00, F01, F02, F03, G30, F051, G311 |
| Chronic pulmonary disease | I278, I279, J40, J41, J42, J43, J44, J45, J46, J47, J60, J61, J62, J63, J64, J65, J66, J67, J684, J701, J703 |
| Rheumatic disease | M05, M06, M315, M32, M33, M34, M351, M353, M360 |
| Peptic ulcer disease | K25, K26, K27, K28 |
| Mild liver disease | B18, K700, K701, K702, K703, K709, K713, K714, K715, K717, K73, K74, K760, K762, K763, K764, K768, K769, Z944 |
| Moderate or severe liver disease | I850, I859, I864, I982, K704, K711, K721, K729, K765, K766, K767 |
| Diabetes without complications | E100, E101, E106, E108, E109, E110, E111, E116, E118, E119, E120, E121, E126, E128, E129, E130, E131, E136, E138, E139, E140, E141, E146, E148, E149 |
| Diabetes with complications | E102, E103, E104, E105, E107, E112, E113, E114, E115, E117, E122, E123, E124, E125, E127, E132, E133, E134, E135, E137, E142, E143, E144, E145, E147 |
| Paraplegia and hemiplegia | G041, G114, G800, G81, G82, G830, G831, G832, G833, G834, G839 |
| Renal disease | I120, I131, N030, N031, N032, N033, N034, N035, N036, N037, N038, N039, N050, N051, N052, N053, N054, N055, N056, N057, N058, N059, N18, N19, N250, Z490, Z491, Z492, Z940, Z992 |
| Any malignancy | C00, C01, C02, C03, C04, C05, C06, C07, C08, C09, C10, C11, C12, C13, C14, C15, C16, C17, C18, C19, C20, C21, C22, C23, C24, C25, C26, C30, C31, C32, C33, C34, C37, C38, C39, C40, C41, C43, C45, C46, C47, C48, C49, C50, C51, C52, C53, C54, C55, C56, C57, C58, C60, C61, C62, C63, C64, C65, C66, C67, C68, C69, C70, C71, C72, C73, C74, C75, C76, C81, C82, C83, C84, C85, C88, C90, C91, C92, C93, C94, C95, C96, C97 |
| Metastatic solid tumor | C77, C78, C79, C80 |
| AIDS/HIV | B20, B21, B22, B24 |
| **Hypertension, uncomplicated** | I10 |
| **Hypertension, complicated** | I11, I12, I13, I15 |

*AIDS* acquired immune deficiency syndrome, *HIV* human immunodeficiency virus, *ICD* International Classification of Diseases

^a^ Hypertension was not included in the Charlson Comorbidity Index, and it was identified separately using the ICD-10 codes

**Table S4** ICD-10-based classification of organ dysfunction

| Variable | Codes |
| --- | --- |
| **Cardiovascular** |  |
| Septic shock | R572 |
| Hypotension | I95 |
| Other hypotension | I958 |
| Hypotension, unspecified | I959 |
| Shock, NEC | R57 |
| Other shock | R578 |
| Shock, unspecified | R579 |
| Shock (endotoxic, hypovolemic) during or following a procedure | T811 |
| Use of a vasopressor (norepinephrine, epinephrine, vasopressin, dopamine, dobutamine) |  |
| **Respiratory** |  |
| Adult respiratory distress syndrome | J80 |
| Pulmonary edema | J81 |
| Respiratory failure, NEC | J96 |
| Acute respiratory failure | J960 |
| Respiratory failure, unspecified | J969 |
| Hypoxemia | R0902 |
| Cyanosis | R230 |
| Dependence on respirator | Z991 |
| Conventional oxygen therapy, high-flow nasal cannula, or mechanical ventilation |  |
| **Neurologic** |  |
| Delirium not induced by alcohol and other psychoactive substances | F05 |
| Other mental disorders due to brain damage and dysfunction and due to physical disease | F06 |
| Organic psychosis NOS | F09 |
| Anoxic brain damage, NEC | G931 |
| Encephalopathy, unspecified | G934 |
| Metabolic encephalopathy | G9380 |
| Somnolence, stupor, and coma | R40 |
| Somnolence | R400 |
| Stupor | R401 |
| Disorientation, unspecified | R410 |
| **Hematologic** |  |
| Disseminated intravascular coagulation (defibrination syndrome) | D65 |
| Other coagulation defects | D68 |
| Other specified coagulation defects | D688 |
| Coagulation defect, unspecified | D689 |
| Purpura and other hemorrhagic conditions | D69 |
| Secondary thrombocytopenia | D695 |
| Thrombocytopenia, unspecified | D696 |
| Spontaneous ecchymoses | R233 |
| Abnormal coagulation lab | R791 |
| **Hepatic** |  |
| Hepatic failure, NEC | K72 |
| Central hemorrhagic necrosis of liver | K762 |
| Infarction of liver | K763 |
| Unspecified jaundice | R17 |
| **Renal** |  |
| Acute renal failure | N17 |
| Unspecified renal failure | N19 |
| Postprocedural renal failure | N990 |
| Anuria and oliguria | R34 |
| Abnormal results of kidney function studies | R944 |
| Dependence on renal dialysis | Z992 |
| Renal replacement therapy |  |
| **Metabolic** |  |
| Acidosis | E872 |

*ICD* International Classification of Diseases, *NEC* not elsewhere classified, *NOS* not otherwise specified

**Table S5** Baseline characteristics of intensive care unit patients in the unmatched cohort

| Characteristics | COVID-19  (n = 28089) | Influenza  (n = 9993) | Control^a^  (n = 11807) | SMD  (COVID-19 vs Influenza) | SMD  (COVID-19 vs Control) |
| --- | --- | --- | --- | --- | --- |
| Age, mean (SD), y | 72.4 (14.4) | 72.7 (14.6) | 71.1 (11.1) | 0.02 | 0.10 |
| Sex, No. (%) |  |  |  | 0.09 | 0.50 |
| Male | 15811 (56.3) | 5192 (52.0) | 9313 (78.9) |  |  |
| Female | 12278 (43.7) | 4801 (48.0) | 2494 (21.1) |  |  |
| Comorbidities, No. (%) |  |  |  |  |  |
| Diabetes | 15381 (54.8) | 4934 (49.4) | 7213 (61.1) | 0.11 | 0.13 |
| Hypertension | 19225 (68.4) | 6775 (67.8) | 8657 (73.3) | 0.01 | 0.11 |
| Myocardial infarction | 1959 (7.0) | 675 (6.8) | 1148 (9.7) | 0.01 | 0.10 |
| Congestive heart failure | 8507 (30.3) | 2662 (26.6) | 4366 (37.0) | 0.08 | 0.14 |
| Cerebrovascular disease | 8861 (31.5) | 2993 (30.0) | 3523 (29.8) | 0.03 | 0.04 |
| Chronic pulmonary disease | 11903 (42.4) | 5606 (56.1) | 7228 (61.2) | 0.28 | 0.38 |
| Chronic liver disease | 11037 (39.3) | 3743 (37.5) | 6544 (55.4) | 0.04 | 0.33 |
| Chronic kidney disease | 4473 (15.9) | 1224 (12.2) | 2531 (21.4) | 0.11 | 0.14 |
| Malignancy | 5961 (21.2) | 1660 (16.6) | 6407 (54.3) | 0.12 | 0.73 |
| Charlson Comorbidity Index, mean (SD) | 3.7 (3.1) | 3.5 (2.8) | 5.8 (3.5) | 0.08 | 0.63 |
| Immunosuppression, No. (%)^b^ | 7541 (26.8) | 2293 (22.9) | 11798 (99.9) | 0.09 | 2.33 |
| Income level, No. (%) |  |  |  |  |  |
| Q1 (lowest) | 7338 (26.1) | 2710 (27.1) | 3072 (26.0) | 0.02 | < 0.001 |
| Q2 | 7041 (25.1) | 2291 (22.9) | 2977 (25.2) | 0.05 | < 0.001 |
| Q3 | 7418 (26.4) | 2635 (26.4) | 2919 (24.7) | < 0.001 | 0.04 |
| Q4 (highest) | 6292 (22.4) | 2357 (23.6) | 2839 (24.0) | 0.03 | 0.04 |
| Hospital size, No. (%) |  |  |  |  |  |
| < 500 beds | 13039 (46.4) | 4807 (48.1) | 3134 (26.5) | 0.03 | 0.41 |
| 500–1000 beds | 12281 (43.7) | 4495 (45.0) | 6064 (51.4) | 0.03 | 0.15 |
| ≥ 1000 beds | 2769 (9.9) | 691 (6.9) | 2609 (22.1) | 0.11 | 0.34 |
| Organ dysfunction, No. (%) |  |  |  |  |  |
| Cardiovascular | 16732 (59.6) | 4721 (47.2) | 6554 (55.5) | 0.25 | 0.08 |
| Respiratory | 25564 (91.0) | 8872 (88.8) | 10130 (85.8) | 0.07 | 0.16 |
| Neurologic | 3378 (12.0) | 927 (9.3) | 817 (6.9) | 0.09 | 0.18 |
| Hematologic | 6132 (21.8) | 878 (8.8) | 1420 (12.0) | 0.37 | 0.26 |
| Hepatic | 828 (2.9) | 152 (1.5) | 254 (2.2) | 0.10 | 0.05 |
| Renal | 6711 (23.9) | 1928 (19.3) | 2542 (21.5) | 0.11 | 0.06 |
| Metabolic | 906 (3.2) | 275 (2.8) | 311 (2.6) | 0.03 | 0.04 |
| Corticosteroids, No. (%) | 18903 (67.3) | 4136 (41.4) | 5363 (45.4) | 0.54 | 0.45 |
| Daily dose, median (IQR), mg^c^ | 53 (45–85) | 65 (33–125) | 64 (34–125) | 0.08 | 0.02 |
| Total days of use, median (IQR) | 8 (4–14) | 4 (1–9) | 3 (1–10) | 0.45 | 0.36 |
| Vasopressor use, No. (%) | 16171 (57.6) | 4560 (45.6) | 6385 (54.1) | 0.24 | 0.07 |
| Oxygen therapy |  |  |  |  |  |
| No oxygen | 2548 (9.1) | 1141 (11.4) | 1685 (14.3) | 0.08 | 0.16 |
| Supplemental oxygen | 8102 (28.8) | 5034 (50.4) | 5699 (48.3) | 0.45 | 0.41 |
| High-flow nasal cannula | 4196 (14.9) | 679 (6.8) | 987 (8.4) | 0.26 | 0.21 |
| Mechanical ventilation | 13243 (47.1) | 3139 (31.4) | 3436 (29.1) | 0.33 | 0.38 |
| Neuromuscular blocking agents, No. (%) | 11150 (39.7) | 2001 (20.0) | 4499 (38.1) | 0.44 | 0.03 |
| Renal replacement therapy, No. (%) | 3300 (11.7) | 655 (6.6) | 1136 (9.6) | 0.18 | 0.07 |
| ECMO, No. (%) | 293 (1.0) | 37 (0.4) | 14 (0.1) | 0.08 | 0.12 |

*COVID-19* coronavirus disease 2019, *ECMO* extracorporeal membrane oxygenation, *IQR* interquartile range, *SD* standard deviation, *SMD* standardized mean difference

^a^ Patient with one of the following: hematological malignancy; allogenic stem-cell transplant; solid organ transplant; use of corticosteroids at ≥ 20 mg/day (prednisolone-equivalent) for ≥ 3 weeks in the past 60 days; treatment with T-cell immunosuppressants, such as calcineurin inhibitors, tumor necrosis factor blockers, lymphocyte-specific monoclonal antibodies, and immunosuppressive nucleoside analogs, during the past 90 days; treatment with inhibitors of B-cell receptor pathway; inherited severe immunodeficiency; or acute graft-versus-host disease

^b^ Immunosuppression included malignancy, human immunodeficiency virus infection, organ transplant, or immunosuppressive therapy

^c^ Methylprednisolone doses and converted doses of dexamethasone and hydrocortisone

**Table S6** Baseline characteristics of high-risk subgroup in the matched cohort

| Characteristics | COVID-19  (n = 3684) | Influenza  (n = 3684) | SMD | COVID-19  (n = 3132) | Control^a^  (n = 3132) | SMD |
| --- | --- | --- | --- | --- | --- | --- |
| Age, mean (SD), y | 71.3 (14.8) | 71.8 (14.5) | 0.03 | 71.2 (11.4) | 71.5 (10.6) | 0.02 |
| Sex, No. (%) |  |  | 0.01 |  |  | 0.05 |
| Male | 2111 (57.3) | 2095 (56.9) |  | 2404 (76.8) | 2472 (78.9) |  |
| Female | 1573 (42.7) | 1589 (43.1) |  | 728 (23.2) | 660 (21.1) |  |
| Comorbidities, No. (%) |  |  |  |  |  |  |
| Diabetes | 1809 (49.1) | 1851 (50.2) | 0.02 | 2049 (65.4) | 2037 (65.0) | < 0.001 |
| Hypertension | 2452 (66.6) | 2468 (67.0) | 0.01 | 2287 (73.0) | 2306 (73.6) | 0.01 |
| Myocardial infarction | 271 (7.4) | 261 (7.1) | 0.01 | 277 (8.8) | 293 (9.4) | 0.02 |
| Congestive heart failure | 1053 (28.6) | 1026 (27.9) | 0.02 | 1251 (39.9) | 1252 (40.0) | < 0.001 |
| Cerebrovascular disease | 1039 (28.2) | 1012 (27.5) | 0.02 | 949 (30.3) | 929 (29.7) | 0.01 |
| Chronic pulmonary disease | 2162 (58.7) | 2199 (59.7) | 0.02 | 2009 (64.1) | 2026 (64.7) | 0.01 |
| Chronic liver disease | 1371 (37.2) | 1390 (37.7) | 0.01 | 1758 (56.1) | 1744 (55.7) | 0.01 |
| Chronic kidney disease | 506 (13.7) | 503 (13.7) | < 0.001 | 741 (23.7) | 717 (22.9) | 0.02 |
| Malignancy | 759 (20.6) | 715 (19.4) | 0.03 | 2224 (71.0) | 2178 (69.5) | 0.03 |
| Charlson Comorbidity Index, mean (SD) | 3.7 (3.2) | 3.6 (2.9) | 0.04 | 6.2 (3.3) | 6.6 (3.7) | 0.10 |
| Immunosuppression, No. (%)^b^ | 1023 (27.8) | 988 (26.8) | 0.02 | 3127 (99.8) | 3128 (99.9) | < 0.001 |
| Income level, No. (%) |  |  |  |  |  |  |
| Q1 (lowest) | 883 (24.0) | 892 (24.2) | 0.01 | 710 (22.7) | 719 (23.0) | 0.01 |
| Q2 | 886 (24.0) | 891 (24.2) | < 0.001 | 804 (25.7) | 785 (25.1) | 0.01 |
| Q3 | 980 (26.6) | 968 (26.3) | 0.01 | 823 (26.3) | 821 (26.2) | < 0.001 |
| Q4 (highest) | 935 (25.4) | 933 (25.3) | < 0.001 | 795 (25.4) | 807 (25.8) | 0.01 |
| Hospital size, No. (%) |  |  |  |  |  |  |
| < 500 beds | 975 (26.5) | 980 (26.6) | < 0.001 | 697 (22.3) | 642 (20.5) | 0.04 |
| 500–1000 beds | 2242 (60.9) | 2265 (61.5) | 0.01 | 1763 (56.3) | 1757 (56.1) | < 0.001 |
| ≥ 1000 beds | 467 (12.7) | 439 (11.9) | 0.02 | 672 (21.5) | 733 (23.4) | 0.05 |
| Organ dysfunction, No. (%) |  |  |  |  |  |  |
| Cardiovascular | 2783 (75.5) | 2712 (73.6) | 0.04 | 2540 (81.1) | 2549 (81.4) | 0.01 |
| Respiratory | 3684 (100) | 3684 (100) |  | 3132 (100) | 3132 (100) | 0.01 |
| Neurologic | 388 (10.5) | 379 (10.3) | 0.01 | 312 (10) | 302 (9.6) | 0.01 |
| Hematologic | 507 (13.8) | 488 (13.2) | 0.01 | 713 (22.8) | 670 (21.4) | 0.03 |
| Hepatic | 59 (1.6) | 53 (1.4) | 0.01 | 117 (3.7) | 104 (3.3) | 0.02 |
| Renal | 1033 (28.0) | 1027 (27.9) | < 0.001 | 1048 (33.5) | 1059 (33.8) | 0.01 |
| Metabolic | 184 (5.0) | 162 (4.4) | 0.03 | 133 (4.2) | 141 (4.5) | 0.01 |
| Corticosteroids, No. (%) | 2434 (61.1) | 2396 (65.0) | 0.02 | 2474 (79.0) | 2422 (77.3) | 0.04 |
| Daily dose, median (IQR), mg^c^ | 53 (49–93) | 72 (40–125) | 0.03 | 56 (51–95) | 71 (40–125) | 0.01 |
| Total days of use, median (IQR) | 8 (4–12) | 5 (1–12) | 0.05 | 10 (5–16) | 6 (2–14) | 0.06 |
| Vasopressor use, No. (%) | 2744 (74.5) | 2674 (72.6) | 0.04 | 2511 (80.2) | 2519 (80.4) | 0.01 |
| Oxygen therapy |  |  |  |  |  |  |
| High-flow nasal cannula | 660 (17.9) | 671 (18.2) | 0.01 | 688 (22.0) | 702 (22.4) | 0.01 |
| Mechanical ventilation | 3024 (82.1) | 3013 (81.8) | 0.01 | 2444 (78.0) | 2430 (77.6) | 0.01 |
| Neuromuscular blocking agents, No. (%) | 1558 (42.3) | 1479 (40.1) | 0.04 | 1663 (53.1) | 1649 (52.7) | 0.01 |
| Renal replacement therapy, No. (%) | 531 (14.4) | 528 (14.3) | < 0.001 | 662 (21.1) | 691 (22.1) | 0.02 |
| ECMO, No. (%) | 42 (1.1) | 34 (0.9) | 0.01 | 20 (0.6) | 14 (0.4) | 0.02 |

*COVID-19* coronavirus disease 2019, *ECMO* extracorporeal membrane oxygenation, *IQR* interquartile range, *SD* standard deviation, *SMD* standardized mean difference

^a^ Patient with one of the following: hematological malignancy; allogenic stem-cell transplant; solid organ transplant; use of corticosteroids at ≥ 20 mg/day (prednisolone-equivalent) for ≥ 3 weeks in the past 60 days; treatment with T-cell immunosuppressants, such as calcineurin inhibitors, tumor necrosis factor blockers, lymphocyte-specific monoclonal antibodies, and immunosuppressive nucleoside analogs, during the past 90 days; treatment with inhibitors of B-cell receptor pathway; inherited severe immunodeficiency; or acute graft-versus-host disease

^b^ Immunosuppression included malignancy, human immunodeficiency virus infection, organ transplant, or immunosuppressive therapy

^c^ Methylprednisolone doses and converted doses of dexamethasone and hydrocortisone

**Table S7** Primary and secondary outcomes of high-risk subgroup

| Outcomes | COVID-19  (n = 3684) | Influenza  (n = 3684) | Difference (95% CI)^a^ | *P* value^b^ | Odds ratio (95% CI) |
| --- | --- | --- | --- | --- | --- |
| **Primary outcome** |  |  |  |  |  |
| Invasive pulmonary aspergillosis, No. (%) | 135 (3.7) | 122 (3.3) | 0.4 (–0.5 to 1.2) | 0.41 | 1.11 (0.87–1.42) |
| **Secondary outcomes** |  |  |  |  |  |
| 30-day mortality, No. (%) | 1370 (37.2) | 1073 (29.1) | 8.1 (5.9 to 10.2) | < 0.001 | 1.44 (1.31–1.59) |
| 180-day mortality, No. (%) | 1991 (54.0) | 1738 (47.2) | 6.9 (4.6 to 9.1) | < 0.001 | 1.32 (1.20–1.44) |
| Ventilator days, median (IQR) | 6 (2–12)  [n = 3024] | 6 (3–13)  [n = 3013] | 0 (0 to 1) | < 0.001 |  |
| Tracheostomy, No. (%) | 370 (10.0) | 451 (12.2) | –2.2 (–3.6 to –0.8) | < 0.001 | 0.80 (0.69–0.93) |
| Length of stay, median (IQR), days |  |  |  |  |  |
| ICU | 7 (3–15) | 8 (4–17) | –1 (–1 to 0) | < 0.001 |  |
| Hospital | 20 (11–38) | 20 (12–36) | 0 (–1 to 0) | 0.16 |  |
| Outcomes | COVID-19  (n = 3132) | Control^c^  (n = 3132) | Difference (95% CI)^a^ | *P* value^b^ | Odds ratio (95% CI) |
| **Primary outcome** |  |  |  |  |  |
| Invasive pulmonary aspergillosis, No. (%) | 247 (7.9) | 220 (7.0) | 0.9 (–0.4 to 2.2) | 0.19 | 1.13 (0.94–1.37) |
| **Secondary outcomes** |  |  |  |  |  |
| 30-day mortality, No. (%) | 1343 (42.9) | 1212 (38.7) | 4.2 (1.8 to 6.6) | < 0.001 | 1.19 (1.08–1.32) |
| 180-day mortality, No. (%) | 2086 (66.6) | 2061 (65.8) | 0.8 (–1.5 to 3.1) | 0.50 | 1.04 (0.93–1.15) |
| Ventilator days, median (IQR) | 7 (3–14)  [n = 2444] | 5 (2–12)  [n = 2430] | 1 (1 to 1) | < 0.001 |  |
| Tracheostomy, No. (%) | 370 (11.8) | 300 (9.6) | 2.2 (0.7 to 3.8) | < 0.001 | 1.27 (1.08–1.49) |
| Length of stay, median (IQR), days |  |  |  |  |  |
| ICU | 7 (3–15) | 6 (2–14) | 1 (1 to 1) | < 0.001 |  |
| Hospital | 22 (12–38) | 22 (13–39) | –1 (–1 to 0) | 0.15 |  |

*CI* confidence interval, *COVID-19* coronavirus disease 2019, *ICU* intensive care unit, *IQR* interquartile range

^a^ Median of all paired differences between the study groups

^b^ *P* values were calculated using the Wilcoxon rank sum test for continuous variables and the chi-square test for categorical variables

^c^ Patient with one of the following: hematological malignancy; allogenic stem-cell transplant; solid organ transplant; use of corticosteroids at ≥ 20 mg/day (prednisolone-equivalent) for ≥ 3 weeks in the past 60 days; treatment with T-cell immunosuppressants, such as calcineurin inhibitors, tumor necrosis factor blockers, lymphocyte-specific monoclonal antibodies, and immunosuppressive nucleoside analogs, during the past 90 days; treatment with inhibitors of B-cell receptor pathway; inherited severe immunodeficiency; or acute graft-versus-host disease

**Table S8** Risk factors for invasive pulmonary aspergillosis in the high-risk subgroup

| Variable | COVID-19 vs Influenza  (n = 3684 vs 3684) | | COVID-19 vs Control^a^  (n = 3132 vs 3132) | |
| --- | --- | --- | --- | --- |
|  | Adjusted odds ratio  (95% CI)^b^ | *P* value | Adjusted odds ratio  (95% CI)^b^ | *P* value |
| Age, y |  |  |  |  |
| 18–39 | 1 (reference) |  | 1 (reference) |  |
| 40–64 | 1.30 (0.66–2.56) | 0.46 | 0.62 (0.20–1.99) | 0.42 |
| ≥ 65 | 1.07 (0.54–2.12) | 0.85 | 0.42 (0.13–1.34) | 0.14 |
| Female sex | 0.81 (0.61–1.07) | 0.13 | 0.62 (0.46–0.85) | 0.003 |
| Comorbidities |  |  |  |  |
| Diabetes | 1.27 (0.94–1.70) | 0.12 | 1.06 (0.84–1.33) | 0.64 |
| Hypertension | 0.74 (0.55–1.01) | 0.06 | 0.84 (0.66–1.07) | 0.16 |
| Myocardial infarction | 0.95 (0.54–1.66) | 0.85 | 0.73 (0.47–1.13) | 0.16 |
| Congestive heart failure | 0.66 (0.46–0.95) | 0.02 | 0.79 (0.62–1.02) | 0.07 |
| Cerebrovascular disease | 0.73 (0.51–1.03) | 0.08 | 0.83 (0.64–1.07) | 0.14 |
| Chronic pulmonary disease | 1.16 (0.86–1.55) | 0.33 | 1.32 (1.05–1.66) | 0.02 |
| Chronic liver disease | 1.32 (0.94–1.86) | 0.11 | 1.06 (0.82–1.36) | 0.67 |
| Chronic kidney disease | 0.98 (0.65–1.47) | 0.91 | 1.05 (0.80–1.37) | 0.72 |
| Malignancy | 1.17 (0.71–1.95) | 0.53 | 1.08 (0.80–1.44) | 0.62 |
| Immunosuppression^c^ | 1.36 (0.86–2.15) | 0.19 | N/A |  |
| Hospital size |  |  |  |  |
| < 500 beds | 1 (reference) |  | 1 (reference) |  |
| 500–1000 beds | 2.16 (1.36–3.43) | 0.001 | 2.08 (1.38–3.15) | 0.001 |
| ≥ 1000 beds | 4.64 (2.78–7.75) | < 0.001 | 4.16 (2.70–6.41) | < 0.001 |
| No. of organ dysfunctions |  |  |  |  |
| 1 | 1 (reference) |  | 1 (reference) |  |
| 2 | 1.44 (0.43–4.77) | 0.55 | 0.79 (0.34–1.85) | 0.58 |
| 3 | 2.23 (0.36–13.83) | 0.39 | 0.77 (0.22–2.70) | 0.68 |
| ≥ 4 | 2.22 (0.17–29.71) | 0.55 | 0.46 (0.08–2.71) | 0.39 |
| Corticosteroids | 3.01 (1.91–4.75) | < 0.001 | 4.28 (2.56–7.18) | < 0.001 |
| Cumulative dose^d^ | 1.00 (1.00–1.00) | 0.07 | 1.00 (1.00–1.00) | 0.31 |
| Total days of use | 1.03 (1.02–1.04) | < 0.001 | 1.03 (1.02–1.03) | < 0.001 |
| Vasopressor use | 0.79 (0.23–2.69) | 0.71 | 1.99 (0.45–8.70) | 0.36 |
| Neuromuscular blocking agents | 1.25 (0.92–1.69) | 0.15 | 1.16 (0.90–1.49) | 0.26 |
| Renal replacement therapy | 1.29 (0.84–1.98) | 0.25 | 1.68 (1.18–2.39) | 0.004 |
| ECMO | 0.79 (0.34–1.85) | 0.59 | 1.43 (0.55–3.73) | 0.47 |
| Length of hospital stay | 1.00 (1.00–1.01) | 0.11 | 1.01 (1.00–1.01) | 0.001 |
| COVID-19 | 1.19 (0.91–1.55) | 0.20 | 1.28 (1.04–1.57) | 0.02 |

*CI* confidence interval, *COVID-19* coronavirus disease 2019, *ECMO* extracorporeal membrane oxygenation, *N/A* not applicable

^a^ Patient with one of the following: hematological malignancy; allogenic stem-cell transplant; solid organ transplant; use of corticosteroids at ≥ 20 mg/day (prednisolone-equivalent) for ≥ 3 weeks in the past 60 days; treatment with T-cell immunosuppressants, such as calcineurin inhibitors, tumor necrosis factor blockers, lymphocyte-specific monoclonal antibodies, and immunosuppressive nucleoside analogs, during the past 90 days; treatment with inhibitors of B-cell receptor pathway; inherited severe immunodeficiency; or acute graft-versus-host disease

^b^ Adjusted for the baseline characteristics listed in Table 1

^c^ Immunosuppression included malignancy, human immunodeficiency virus infection, organ transplant, or immunosuppressive therapy

^d^ Methylprednisolone doses and converted doses of dexamethasone and hydrocortisone

**Table S9** Primary and secondary outcomes according to steroid dose and duration^a^

| COVID-19 (n = 18903) | | | | | |
| --- | --- | --- | --- | --- | --- |
| Outcomes | Higher dose and longer duration  (n = 8213) | Higher dose and shorter duration  (n = 4909) | Lower dose and longer duration  (n = 3151) | Lower dose and shorter duration  (n = 2630) | *P* value^b^ |
| **Primary outcome** |  |  |  |  |  |
| Invasive pulmonary aspergillosis, No. (%) | 657 (8.0) | 71 (1.4) | 223 (7.1) | 25 (1.0) | < 0.001 |
| **Secondary outcomes** |  |  |  |  |  |
| 30-day mortality, No. (%) | 2964 (36.1) | 1602 (32.6) | 920 (29.2) | 615 (23.4) | < 0.001 |
| 180-day mortality, No. (%) | 4770 (58.1) | 2299 (46.8) | 1633 (51.8) | 1071 (40.7) | < 0.001 |
| Ventilator days, median (IQR) | 11 (6–19)  [n = 5863] | 4 (2–8)  [n = 2305] | 11 (6–22)  [n = 2091] | 4 (2–9)  [n = 1022] | < 0.001 |
| Tracheostomy, No. (%) | 1180 (14.4) | 233 (4.7) | 513 (16.3) | 110 (4.2) | < 0.001 |
| Length of stay, median (IQR), days |  |  |  |  |  |
| ICU | 9 (5–18) | 5 (2–10) | 9 (4–19) | 5 (2–11) | < 0.001 |
| Hospital | 23 (14–40) | 13 (7–29) | 24 (15–42) | 21 (9–45) | < 0.001 |
| Influenza (n = 4136) | | | | | |
| Outcomes | Higher dose and longer duration  (n = 988) | Higher dose and shorter duration  (n = 1453) | Lower dose and longer duration  (n = 456) | Lower dose and shorter duration  (n = 1239) | *P* value^b^ |
| **Primary outcome** |  |  |  |  |  |
| Invasive pulmonary aspergillosis, No. (%) | 52 (5.3) | 25 (1.7) | 32 (7.0) | 23 (1.9) | < 0.001 |
| **Secondary outcomes** |  |  |  |  |  |
| 30-day mortality, No. (%) | 235 (23.8) | 331 (22.8) | 86 (18.9) | 194 (15.7) | < 0.001 |
| 180-day mortality, No. (%) | 449 (45.4) | 530 (36.5) | 191 (41.9) | 379 (30.6) | < 0.001 |
| Ventilator days, median (IQR) | 9 (5–18)  [n = 609] | 5 (2–11)  [n = 673] | 10 (5–22)  [n = 279] | 6 (3–12)  [n = 523] | < 0.001 |
| Tracheostomy, No. (%) | 126 (10.2) | 80 (5.5) | 73 (16.0) | 70 (5.6) | < 0.001 |
| Length of stay, median (IQR), days |  |  |  |  |  |
| ICU | 8 (4–17) | 4 (2–9) | 9 (4–22) | 5 (2–11) | < 0.001 |
| Hospital | 24 (16–40) | 16 (9–28) | 27 (16–44) | 19 (11–33) | < 0.001 |
| Control (n = 5363)^c^ | | | | | |
| Outcomes | Higher dose and longer duration  (n = 1311) | Higher dose and shorter duration  (n = 1994) | Lower dose and longer duration  (n = 548) | Lower dose and shorter duration  (n = 1510) | *P* value^b^ |
| **Primary outcome** |  |  |  |  |  |
| Invasive pulmonary aspergillosis, No. (%) | 140 (10.7) | 63 (3.2) | 66 (12.0) | 35 (2.3) | < 0.001 |
| **Secondary outcomes** |  |  |  |  |  |
| 30-day mortality, No. (%) | 377 (28.8) | 593 (29.7) | 133 (24.3) | 279 (18.5) | < 0.001 |
| 180-day mortality, No. (%) | 775 (59.1) | 963 (48.3) | 327 (59.7) | 586 (38.8) | < 0.001 |
| Ventilator days, median (IQR) | 9 (4–15)  [n = 743] | 3 (2–16)  [n = 821] | 10 (5–23)  [n = 306] | 3 (2–8)  [n = 490] | < 0.001 |
| Tracheostomy, No. (%) | 125 (9.5) | 54 (2.7) | 76 (13.9) | 45 (3.0) | < 0.001 |
| Length of stay, median (IQR), days |  |  |  |  |  |
| ICU | 7 (3–15) | 2 (1–5) | 8 (4–20) | 2 (1–6) | < 0.001 |
| Hospital | 27 (18–40) | 16 (9–27) | 31 (20–52) | 16 (10–28) | < 0.001 |

*COVID-19* coronavirus disease 2019, *ICU* intensive care unit, *IQR* interquartile range

^a^ Stratified by methylprednisolone dose of ≥ 50 or < 50 mg and duration of ≥ 7 or < 7 days

^b^ *P* values were calculated using the Kruskal–Wallis test for continuous variables and the chi-square test for categorical variables

^c^ Patient with one of the following: hematological malignancy; allogenic stem-cell transplant; solid organ transplant; use of corticosteroids at ≥ 20 mg/day (prednisolone-equivalent) for ≥ 3 weeks in the past 60 days; treatment with T-cell immunosuppressants, such as calcineurin inhibitors, tumor necrosis factor blockers, lymphocyte-specific monoclonal antibodies, and immunosuppressive nucleoside analogs, during the past 90 days; treatment with inhibitors of B-cell receptor pathway; inherited severe immunodeficiency; or acute graft-versus-host disease

**Table S10** Risk factors for COVID-19-associated pulmonary aspergillosis

| Variable | No. of cases/patients  (%) | Unadjusted odds ratio  (95% CI) | Adjusted odds ratio  (95% CI)^a^ |
| --- | --- | --- | --- |
| Age, y |  |  |  |
| 18–39 | 26/879 (3.0) | 1 (reference) | 1 (reference) |
| 40–64 | 288/6337 (4.5) | 1.56 (1.04–2.35) | 1.47 (0.93–2.35) |
| ≥ 65 | 713/20873 (3.4) | 1.16 (0.78–1.73) | 1.52 (0.96–2.41) |
| Female sex | 363/12278 (3.0) | 0.70 (0.61–0.79) | 0.92 (0.80–1.06) |
| Charlson Comorbidity Index |  |  |  |
| 0–1 | 285/7134 (4.0) | 1 (reference) | 1 (reference) |
| 2–3 | 271/8208 (3.3) | 0.82 (0.69–0.97) | 0.81 (0.65–0.99) |
| ≥ 4 | 471/12747 (3.7) | 0.92 (0.79–1.07) | 0.87 (0.65–1.15) |
| Immunosuppression^b^ | 401/7541 (5.3) | 1.79 (1.57–2.03) | 1.79 (1.43–2.24) |
| Hospital size |  |  |  |
| < 500 beds | 167/13039 (1.3) | 1 (reference) | 1 (reference) |
| 500–1000 beds | 577/12281 (4.7) | 3.80 (3.19–4.52) | 2.00 (1.66–2.41) |
| ≥ 1000 beds | 283/2769 (10.2) | 8.78 (7.21–10.68) | 3.33 (2.67–4.16) |
| No. of organ dysfunctions |  |  |  |
| 1 | 61/8208 (0.7) | 1 (reference) | 1 (reference) |
| 2 | 367/10020 (3.7) | 5.08 (3.87–6.67) | 1.27 (0.76–2.12) |
| 3 | 363/6663 (5.4) | 7.69 (5.85–10.11) | 1.13 (0.51–2.47) |
| ≥ 4 | 236/3198 (7.4) | 10.64 (8.00–14.14) | 1.05 (0.33–3.32) |
| Corticosteroids |  |  |  |
| Cumulative dose^c^ |  | 1.00 (1.00–1.00) | 1.00 (1.00–1.00) |
| Total days of use |  | 1.05 (1.05–1.06) | 1.03 (1.03–1.04) |
| Tocilizumab | 129/1509 (8.5) | 2.67 (2.21–3.24) | 1.48 (1.20–1.83) |
| Baricitinib | 105/769 (13.7) | 4.53 (3.65–5.62) | 2.47 (1.93–3.15) |
| Vasopressor use | 914/16171 (5.7) | 6.26 (5.14–7.62) | 3.29 (1.20–8.97) |
| Renal replacement therapy | 302/3300 (9.2) | 3.34 (2.91–3.84) | 1.39 (1.09–1.76) |
| ECMO | 54/293 (18.4) | 6.23 (4.60–8.43) | 1.09 (0.77–1.56) |

*CI* confidence interval, *COVID-19* coronavirus disease 2019, *ECMO* extracorporeal membrane oxygenation

^a^ The full model (i.e., all included covariates) is shown

^b^ Immunosuppression included malignancy, human immunodeficiency virus infection, organ transplant, or immunosuppressive therapy

^c^ Methylprednisolone doses and converted doses of dexamethasone and hydrocortisone

**Table S11** Incidence of CAPA according to the vaccination status and study period

| Variable | CAPA incidence (no. of patients/total no. [%]) | | |
| --- | --- | --- | --- |
|  | Unmatched  (n = 28089) | Influenza-matched  (n = 9649) | Control-matched  (n = 5709) |
| Vaccination status |  |  |  |
| Unvaccinated | 513/9609 (5.3) | 86/2849 (3.0) | 90/1595 (5.6) |
| Vaccinated | 514/18480 (2.8) | 104/6800 (1.5) | 185/4114 (4.5) |
| Study period |  |  |  |
| Before Omicron (20.10–21.12) | 475/6379 (7.4) | 75/1697 (4.4) | 76/911 (8.3) |
| Omicron (22.1–22.10) | 552/21710 (2.5) | 115/7952 (1.4) | 199/4798 (4.1) |

*CAPA* COVID-19-associated pulmonary aspergillosis
